# Reducing PM_10_ and PM_2.5_ Concentrations in a Subway Station by Changing the Diffuser Arrangement

**DOI:** 10.3390/toxics10090537

**Published:** 2022-09-15

**Authors:** Seong-Gyu Kim, Gibong Sung, Se-Jin Yook, Minjeong Kim, Duckshin Park

**Affiliations:** 1 School of Mechanical Engineering, Hanyang University, Seoul 04763, Korea; 2 Artificial Intelligence Railroad Research Department, Korea Railroad Research Institute, Uiwang-si 17104, Gyeonggi-do, Korea; 3 Transportation Environmental Research Department, Korea Railroad Research Institute, Uiwang-si 16105, Gyeonggi-do, Korea

**Keywords:** particulate matter, PM_10_, PM_2.5_, subway station, indoor air quality, diffuser arrangement

## Abstract

According to the stringent regulations on particulate matter (PM) concentrations in Seoul, Korea, the PM_10_ and PM_2.5_ concentrations in subway stations must be maintained below 50 and 30 μg/m^3^, respectively, by 2024. Therefore, the PM concentrations in a subway station were analyzed considering air-conditioning diffuser arrangement and filtration efficiency, with the total ventilation flow rate of the station maintained constant. Dynamic analysis was performed under a worst-case scenario, wherein outdoor air was introduced through ground entrances and high-concentration dust (PM_10_, PM_2.5_) was introduced from stationary train cabins into the platforms through open platform screen doors (PSDs). Although the average PM concentrations were predicted to satisfy the reinforced criteria of Seoul under the existing operating conditions, the recommended limits were exceeded in certain local areas. To address this, the PM concentrations were predicted by changing the diffuser arrangement in the waiting room and maintaining the total ventilation flow rate constant. When the diffusers were placed near the waiting room walls, the PM_10_ and PM_2.5_ concentrations were reduced by approximately 10.5 and 5%, respectively, compared to the previous diffuser arrangement. Thus, the required PM concentration criteria were satisfied in nearly all areas of the target station, except for certain areas close to PSDs. The study findings can form the basis for improving the air quality of other subway stations.

## 1. Introduction

Subways are one of the most popular means of transportation, with the number of subway commuters steadily increasing over the years. In 2016, subway users accounted for 59.3% of the public transportation users in Seoul, Korea [1]. While the number of bus users decreased by 108,000, the number of subway users increased by 14,000 [2]. This increase in the number of subway users has expanded the interest in indoor air quality management in the subway environment. Previous studies have reported that the particulate matter (PM) concentrations in subway stations are generally higher than those in the atmosphere [3,4]. As subway stations are located underground, passengers can be easily exposed to toxic pollutants, including PM. Furthermore, a study reported that among subway, bicycle, bus, and walking commuters, subway commuters are exposed to the highest PM_2.5_ concentration [5]. PM is considered hazardous and can adversely affect human health by penetrating the lungs owing to its small size [6]. Exposure to PM is a major risk that reduces life expectancy and causes various diseases [7]. It can aggravate airway inflammation, asthma and other allergic diseases [8], increase cardiovascular diseases and mortality [9], cause neurodevelopmental disorders for fetuses in pregnant women [10], and increase the possibility of autism spectrum disorders [11].

Therefore, stringent PM regulations have been introduced in Seoul, Korea; accordingly, the PM concentration criteria for subway stations must be maintained below 50 and 30 μg/m^3^ for PM_10_ and PM_2.5_ by 2024, respectively. The recently measured PM concentrations in the subway environment of Seoul were 111 μg/m^3^ for PM_10_ and 58 μg/m^3^ for PM_2.5_, which significantly exceed the required criteria [12]. Typically, air inflow from underground tunnels, contaminated cabins, and station entrances along with the influence of aging stations are the factors that affect PM concentrations in subway stations. Several studies have actively focused on reducing PM concentrations in the subway environment. Countries such as Korea, Brazil, Canada, Denmark, Japan, the UK, and the US have installed platform screen doors (PSDs) to reduce the risk of train accidents and prevent the inflow of PM from underground tunnels [13]. Woo et al. [14] tested the performance of six types of electrostatic precipitators (ESPs) in a lab-scale wind tunnel; they reported that the saw-type ESP was the most suitable for particle removal in subway tunnels, as it exhibited a collection efficiency of 90% under the 1.5-W/(m/s) condition and the lowest ozone emission rate (16.4 μg/s per mA). Lee et al. [15] developed high-efficiency ESPs for air-conditioners in subway stations, which contributed to the improvement of air quality and energy efficiency by achieving higher-grade filters than the existing ones used in subway station air-conditioners. Wang et al. [16] evaluated the holistic performance of commercially available duct-type ESPs; they concluded that the overall efficiencies of the ESPs with dielectric coatings were comparable to those of the F7 grade filters. Afshari et al. [17] reviewed the literature on ESPs and gave overview of the use of the ESPs as indoor air cleaners. Furthermore, Lee et al. [18] analyzed the influence of the piston effect of a train traveling in a subway tunnel on the ventilation flow rate using computational fluid dynamics (CFD); they reported that vents placed at a distance of approximately 50 m from the platforms with PSDs are desirable for the natural ventilation system. Lee et al. [19] used CFD to analyze the behaviors of particles generated by the friction between the wheels of a moving subway train and the tracks, as well as those re-scattered from the tunnel floor by the train-induced wind; they suggested that the space under a T-car (trailer vehicle without the driver’s seat) is suitable for the installation of precipitators because numerous particles move under the T-car and the airflow in this space is relatively uniform compared to other places on a train. Liu et al. [20] investigated the natural ventilation effect of horizontal entrances in subway stations using CFD and the wind tunnel test. They confirmed that the proportion of the positive pressure area in the horizontal opening can be effectively increased by increasing the height of the shelter, whereas the turbulence of the opening can be increased by increasing the shelter length. Tao et al. [21] employed the CFD technique to predict the performance of ventilation holes in subway cabin ventilation panels using porous media and porous jump. They reported that the developed CFD technique was cost-effective in predicting the flow in a subway cabin. Bolourchi et al. [22] analyzed the PM concentration level in a subway station in Tehran, and proposed optimization measures to satisfy the respiratory health requirements of workers and passengers. Teodosiu et al. [23] conducted CFD investigations on ventilation efficiency in the case of a train fire in a subway tunnel and examined the possibility of preventing the exposure of the ventilation efficiency of the subway station to high-temperature or high-concentration carbon monoxide interference under emergency evacuation conditions. Song et al. [24] analyzed the spatial distribution characteristics of PM_2.5_ in the long underground passage of Shanghai South Railway Station, considering the inlet position and air velocity using the CFD method; they proposed a method to improve the air quality inside the underground passage.

Most existing studies predominantly focus on the concentration and spatial distribution characteristics of PM in subway environments, such as stations, tunnels, and train cabins. However, studies related to the influence of the overall vent arrangement in a station and the filtration efficiency on the air quality within the station are insufficient. Ideally, PM can be removed through the filters used in the air-handling unit (AHU) of the heating, ventilation, and air conditioning system. Moreover, cost-effectively achieving accurate filtration efficiency is essential for reducing the indoor PM concentration [25]. In this study, the airflow in a subway station in Korea was analyzed using CFD, and the PM_2.5_ and PM_10_ concentrations were predicted by changing the inlet/outlet arrangement, the flow rate of the ventilation system, and the filtration efficiency. The developed numerical method was used to measure the ventilation in the station, and the obtained results verified that the stringent PM regulations of Seoul were satisfied.

## 2. Numerical Method

**Figure 1:**
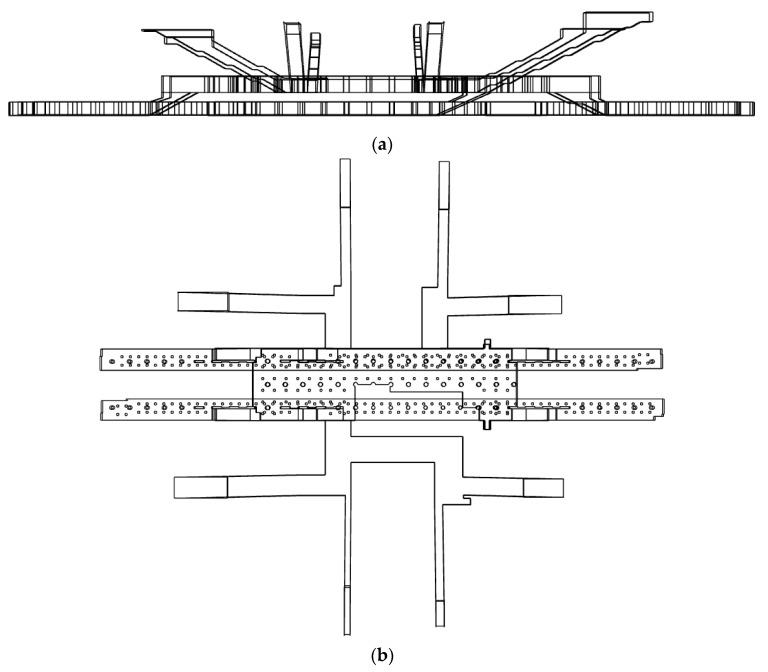
Schematic of the target subway station: (**a**) Side view; (**b**) Top view.

Station Y in the Seoul metropolitan area of Korea was selected as the target station for the analysis; Figure 1 depicts the schematic of the station. The station includes several zones, such as air-conditioning rooms, restrooms, and station offices; however, only the underground waiting room and the platforms used by general passengers are considered as targets for the analysis in this study. The station is 161.6 m long, 25.6 m wide, and 22 m high, with a typical structure of two underground floors and no transit zone. The waiting room is located at a depth of 14.8 m from the ground, and the platforms are at a depth of 19.8 m from the waiting room. The station has eight external entrances connected to the hallways from the waiting room. On the platforms, the lines are separated in opposite directions. The platforms in opposite directions face each other with train tracks located in the middle. Owing to the installed PSDs, the platforms are spatially separated from the traveling trains. Although PSDs are normally closed, they remain open during the arrival of trains for the boarding and deboarding of passengers.

**Figure 2:**
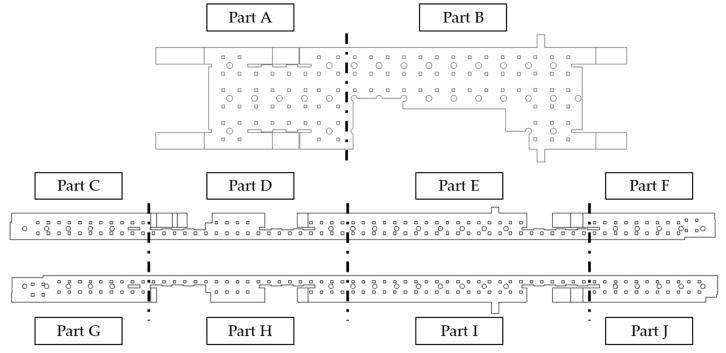
Diffuser arrangement in each air-conditioning part of station Y (target station of the analysis).

Figure 2 depicts the arrangement of the circular diffusers in each part of the subway station. The small squares (□) and circles (○) indicate the positions of diffusers and columns, respectively. Considering the position of the smoke reservoir screen and the arrangement of AHU, the first basement floor with the waiting room was divided into two parts, namely, A and B, whereas the second basement floor with the platforms was divided into eight parts, C to J. Table 1 lists the number of supply air (SA) and return air (RA) diffusers used in each part and the airflow rate through each diffuser. Part A on the first basement floor was connected to the hallways in the up and down directions; therefore, the air was supplied at a higher flow rate than that in part B for efficient air circulation. Furthermore, 30% of the air sucked through the RA diffusers was discharged to the outside of the station, whereas the remaining 70% was combined with the 30% airflow that was newly introduced from the ground into the station through filters and SA diffusers.

ANSYS FLUENT *Release 19.0* was used to analyze the airflow and PM concentration distribution in the target station. The airflow was assumed to be three-dimensional, steady, incompressible, and turbulent. The continuity and momentum equations were solved for the flow analysis [22,26,27]. The standard *k–ε* turbulence model was used for the turbulence analysis based on several previously reported studies that analyzed the airflow in the subway environment [28,29,30,31]. For flow analysis, the Quadratic Upstream Interpolation for Convective Kinematics (QUICK) was selected as the momentum equation scheme, the Semi-Implicit Method for Pressure Linked Equations (SIMPLE) as the pressure−velocity coupling scheme, and the Pressure Staggering Option (PRESTO) as the pressure interpolation scheme. As the diffuser size was considerably small compared to the size of the station and the air supplied from the diffusers flowed along the ceiling surface, the space including the geometry of one diffuser and the surrounding ceiling was modeled separately on a small scale. Additionally, the velocity profile of the air supplied from the diffuser was obtained for each flow rate. The boundary conditions of the flow analysis can be summarized as follows:

1. The velocity inlet condition was applied to SA diffusers, and the velocity profile acquired in advance according to the flow rate was applied to each diffuser;
2. The pressure outlet condition was applied to RA diffusers, and the target mass flow rate was set for the RA flow rate to be sucked through each diffuser;
3. The no-slip condition was applied to all walls in the station, including ceilings, floors, and columns;
4. The no-slip condition was applied to the walls and entrances of PSDs when they were closed, whereas the pressure outlet condition was applied to the entrances of PSDs when they were opened. This aided in implementing a scenario where the air flowed owing to the pressure difference between the platform and the cabins of a stationary train;
5. Finally, the scenario of air flowing because of the pressure difference between the ground and underground station was implemented by setting the pressure outlet at the station entrances on the ground. The temperature and pressure in the computation domain were set to 20 °C and 101.3 kPa, respectively.

PM concentration transport was analyzed based on the Eulerian approach, i.e., the User-Defined Scalar transport equation (Equation (1)) was solved using the FLUENT [32]:

(1) $$\frac{\partial \rho \phi }{\partial t}\;+\;\frac{\partial }{\partial x_{i}}\left(\rho u_{i}\phi \;-\;\Gamma \frac{\partial \phi }{\partial x_{i}}\right)\;=\;S_{\phi }$$

where ϕ is the PM concentration, *ρ* is the air density, *t* is the time, $x_{i}$ is the coordinate, $u_{i}$ is the fluid velocity, Γ is the diffusion coefficient, and $S_{\phi }$ is the source term. The PM concentration distribution was analyzed using a user-defined function considering the aforementioned ventilation method and the particle removal efficiency of the filters. In other words, by considering the flow rate ratios of return air and outdoor air for each AHU, 70% of the average value of the PM concentrations at the RA diffusers was added to 30% of the outdoor PM concentration, and then this sum was multiplied by ‘(1—filtration efficiency)’ to set the PM concentration at the SA diffusers. The outdoor PM concentrations above the ground were measured and reflected in the simulation. Accordingly, the PM_2.5_ and PM_10_ concentrations were set to 22 and 45 μg/m^3^, respectively. The moment at which the PM concentrations in the station reached their peaks during the operation of the ventilation system was assumed to be the time when a subway train halted at the station, causing the air to flow from the cabins to the platforms. To simulate this situation, the airflow and PM concentrations in the station were initially analyzed considering a steady-state condition with the PSDs closed. The PM_2.5_ and PM_10_ concentrations in the station were acquired after 20 s by analyzing the situation of PSDs being opened for 20 s and air flowing between the subway cabins and platforms in a transient state. The PM concentrations measured in the subway cabin were reflected in the simulation, wherein the PM_2.5_ and PM_10_ concentrations were set to 22 and 105 μg/m^3^, respectively.

**Figure 3:**
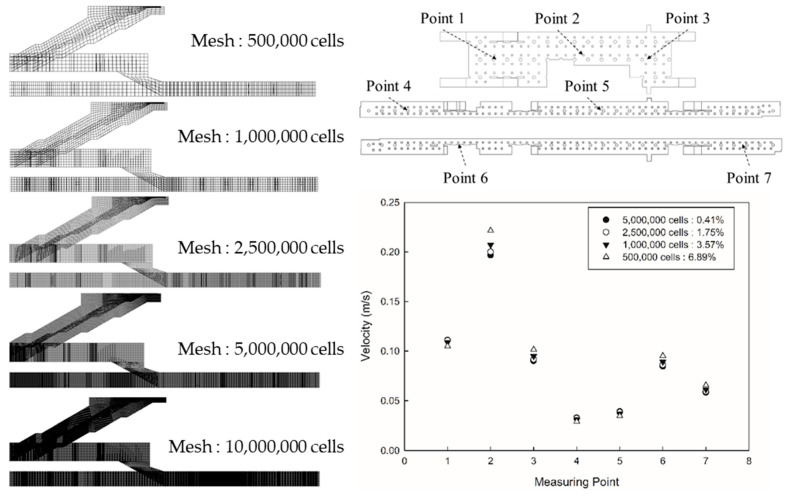
Mesh levels for the grid independence test and the subsequent flow velocity distributions.

The grid independence test was performed by changing the number of meshes from approximately 0.5 to 10 million, based on a method reported in previous studies [33,34] (Figure 3). The convergence condition of all equations for flow and PM concentration analysis was set to 10^−5^. The boundary conditions applied to each mesh level were identical to those used earlier. As indicated in the figure, the velocities were compared at seven arbitrary points; three points in the waiting room and four points on the platforms. The comparison based on the largest number of meshes (10 million) indicated that the error decreased with the increase in the number of meshes. When the number of meshes was approximately 5 million, the error was 0.41%. Therefore, the number of meshes was set to 5,446,743 for the analysis considering the calculation efficiency and accuracy.

**Figure 4:**
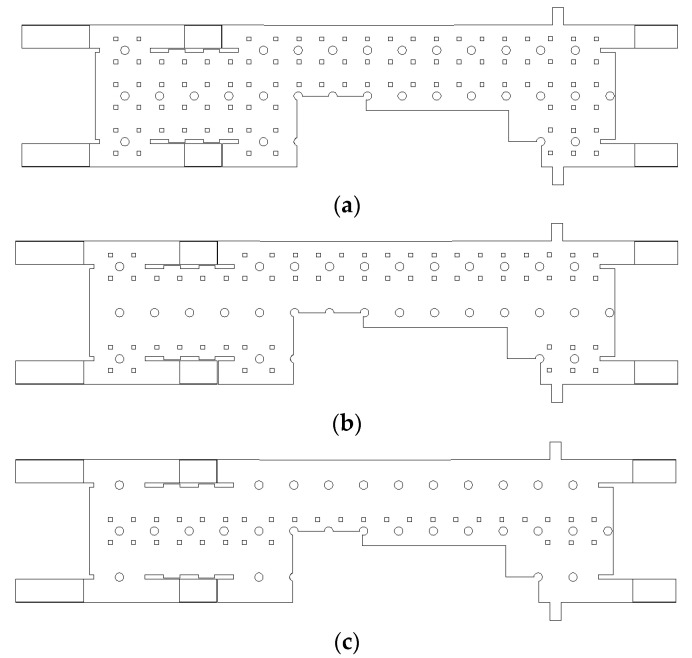
Diffuser arrangement types in the waiting room (floor B1): (**a**) type A: reference; (**b**) type B: diffuser arrangement near the walls; (**c**) type C: diffuser arrangement in the central area.

**Figure 5:**
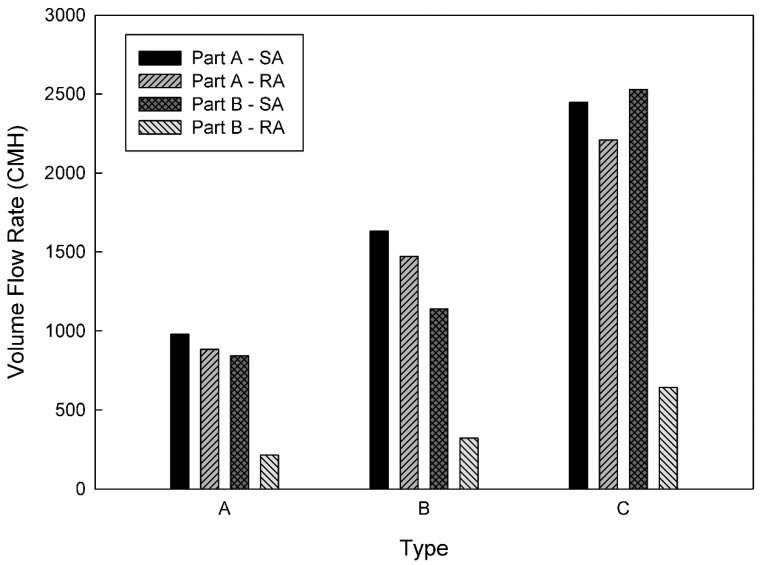
Comparison of the flow rates of individual supply air (SA) and return air (RA) diffusers in each part of the waiting room (floor B1) according to different types of diffuser arrangements.

The objective of this study was to propose an air-conditioning system that can satisfy the stringent PM concentration criteria of Seoul for subway stations by rearranging the diffusers in the station, adjusting the flow rate, and changing the filtration efficiency based on the existing air-conditioning system and filtration efficiency. To this end, three types of diffuser arrangement models were considered for the waiting room on the first basement floor, as indicated in Figure 4. Type A model represents the current positions of diffusers in the waiting room of station Y. Type B model represents the scenario with the diffusers in the middle of the passage removed, and the flow rate of the diffusers increased near both walls. Type C model represents the scenario with the diffusers near both walls removed, and the flow rate of the diffusers increased in the middle of the passage. The same total ventilation flow rate was applied to all three types, and the flow rate of each diffuser was set to the value obtained by dividing the total ventilation flow rate by the number of diffusers. Figure 5 depicts a comparison of the SA and RA flow rates in parts A and B of the waiting room for each type. In the case of types B and C, the number of SA diffusers were decreased to 32 and 17, whereas the number of RA diffusers were decreased to 28 and 16, respectively. Therefore, type C exhibited the highest flow rate in each diffuser, followed by types B and A. Additionally, the filtration efficiency was increased from 70 to 80%, which then reached 90%. Table 2 summarizes the nine cases considered in this study according to the combination of each diffuser arrangement type and filtration efficiency.

## 3. Results and Discussion

**Figure 6:**
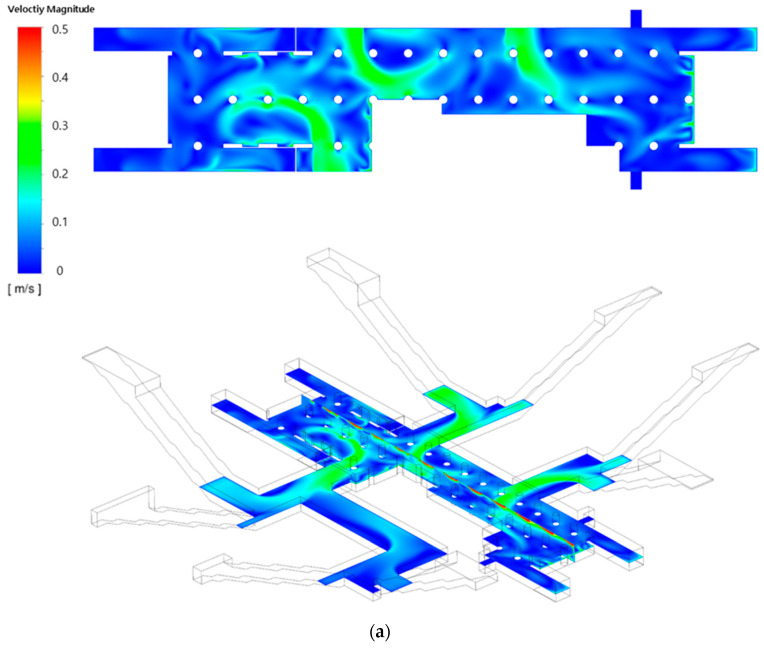

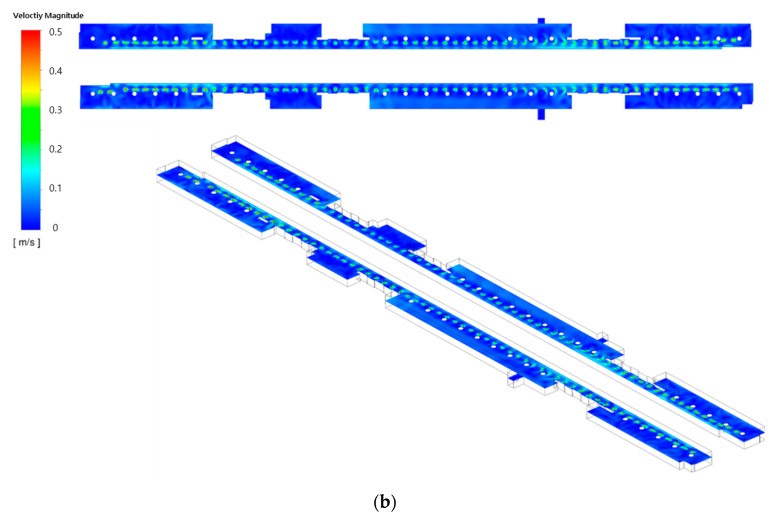
Airflow velocity distributions under the application of the current air-conditioning system of station Y (Case 1): (**a**) waiting room (floor B1); (**b**) platforms (floor B2).

Figure 6a,b depict the airflow velocity distributions in the waiting room on the first basement floor and the platforms on the second basement floor, respectively, considering the type A diffuser arrangement and operating flow rate (Table 1) of the current air-conditioning system of station Y. The velocity distribution in the vertical plane observed in the isometric view (Figure 6a) confirmed that the air introduced through each SA diffuser moved along the ceiling at a high speed and then diffused to lower areas, thereby generating a relatively low flow velocity at the height of the breathing line. Furthermore, the air flowed from the ground entrances to the waiting room. As the left side (part A) of the waiting room was connected to six ground entrances and the wind was blown from both sides, more complex flow patterns were observed than those on the right side (part B), which was connected to two ground entrances with the wind blowing from only one side. The velocity distribution on the second basement floor (Figure 6b) indicated that the flow velocity was generally low in comparison with that of the first basement floor. Moreover, the air flowed from the subway cabins toward the platforms through certain areas where PSDs were opened because the train was stationary.

**Figure 7:**
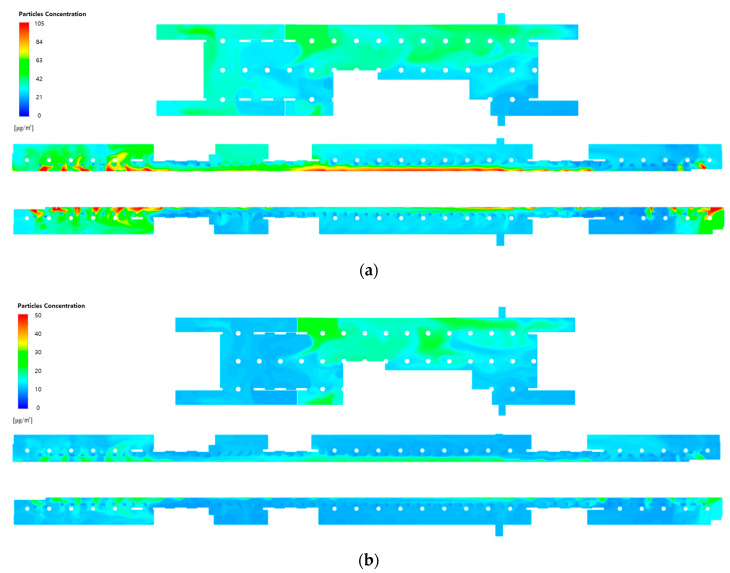
Particulate matter (PM) concentration contours in the waiting room (floor B1) and platforms (floor B2) under the application of the current air-conditioning system of station Y (Case 1): (**a**) PM_10_ concentration; (**b**) PM_2.5_ concentration.

Figure 7 depicts the PM_10_ and PM_2.5_ concentration distributions at the height of the breathing line in the waiting room and platforms when the type A diffuser arrangement and operating flow rate (Table 1) of the current air-conditioning system of station Y are applied. Here, the filtration efficiency was assumed to be 70%, which represents Case 1. In the waiting room, the average PM_10_ concentration was predicted to be 33.5 μg/m^3^ at the height of the breathing line and 32.7 μg/m^3^ in the entire space; conversely, the average PM_2.5_ concentration was predicted to be 12.0 μg/m^3^ at the height of the breathing line and 11.8 μg/m^3^ in the entire space. In general, the average PM concentrations at the height of the breathing line were slightly higher than those in the entire space because the relatively clean air introduced through the diffusers initially flowed along the ceiling before descending. As the airflow introduced through the hallways connected to the ground entrances was mixed with the airflow generated by the air-conditioning system resulting in complex flow patterns, the PM_10_ and PM_2.5_ concentration distributions in the waiting room significantly varied depending on the space. The PM concentrations were high in the areas connected to the hallways because the airflow was introduced from the ground entrances. However, the concentrations were higher in the areas close to the stairs connected to the second basement floor owing to the airflow introduced from the platforms. In the case of the platforms, the average PM_10_ concentration was 36.4 μg/m^3^ at the height of the breathing line and 35.2 μg/m^3^ in the entire space, whereas the average PM_2.5_ concentration was 14.2 μg/m^3^ at the height of the breathing line and 13.9 μg/m^3^ in the entire space. Overall, the PM concentrations on the second basement floor were slightly higher than those on the first basement floor. This is because air with high PM concentrations flowed from the subway cabins toward the platforms when the PSDs were opened; moreover, the number of diffusers was relatively small compared to the large space. As the height of the PSDs (2 m) was lower than the ceiling height of the second basement floor, the average concentrations at the height of the breathing line were higher than those in the entire space. Although the platforms facing each other were symmetrically structured, the PM concentration distribution was different between the platforms. This is because the concentration distribution was affected by the flow pattern of the first basement floor as the platforms were connected to this floor through stairs. Both PM_10_ and PM_2.5_ concentrations exhibited a tendency to decrease as the distance from the PSDs increased. This can be attributed to the relatively clean airflow supplied from the SA diffusers, which were located immediately in front of the PSDs, serving as an air curtain and slightly blocking the airflow introduced from the subway cabins. Furthermore, relatively high PM concentrations were observed at both ends of the platforms. This is because although the airflow with high PM concentrations was introduced through the opened PSDs at both platform ends, no diffuser was placed in the outer areas of the pillars at these platform ends.

**Figure 8:**
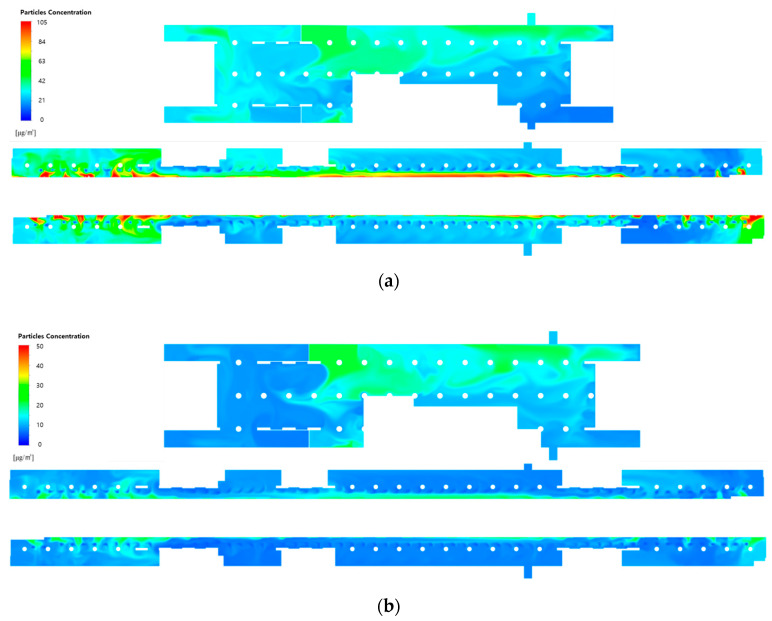

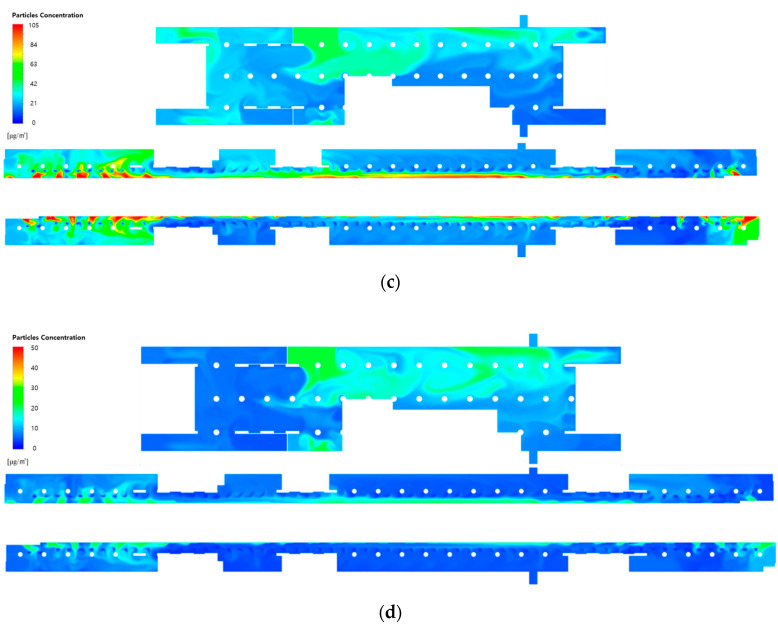
PM concentration contours in the waiting room (floor B1) and platforms (floor B2) according to the filtration efficiency of the current air-conditioning system of station Y: (**a**) PM_10_ and (**b**) PM_2.5_ concentrations at a filtration efficiency of 80% (Case 2); (**c**) PM_10_ and (**d**) PM_2.5_ concentrations at a filtration efficiency of 90% (Case 3).

Figure 8 shows the PM_10_ and PM_2.5_ concentration distributions at the height of the breathing line in the waiting room and platforms when the filtration efficiency was increased to 80% (Case 2) and 90% (Case 3), while the type A diffuser arrangement and operating flow rate (Table 1) of the current air-conditioning system of station Y were applied. A comparison with the results of applying a filtration efficiency of 70% (Figure 7) indicated that the PM concentrations decreased in both the waiting room and platforms with the increase in filtration efficiency. In the case of the waiting room, the reduction in the PM concentration was insignificant in the areas that led to the hallways connected to the ground entrances. This is because the airflow was introduced from the ground. In the case of the platforms, the PM concentrations were considerably high in the vicinity of opened PSDs because the airflow was introduced from the subway cabins. Additionally, the PM concentrations were determined to be high at both ends of the platforms despite the absence of diffusers, because the effect of improving the filter grade was insignificant. This indicated that increasing the filtration efficiency might reduce the average PM concentrations in the subway station; nevertheless, the local areas were excluded, wherein the air quality improvement was insignificant.

**Figure 9:**
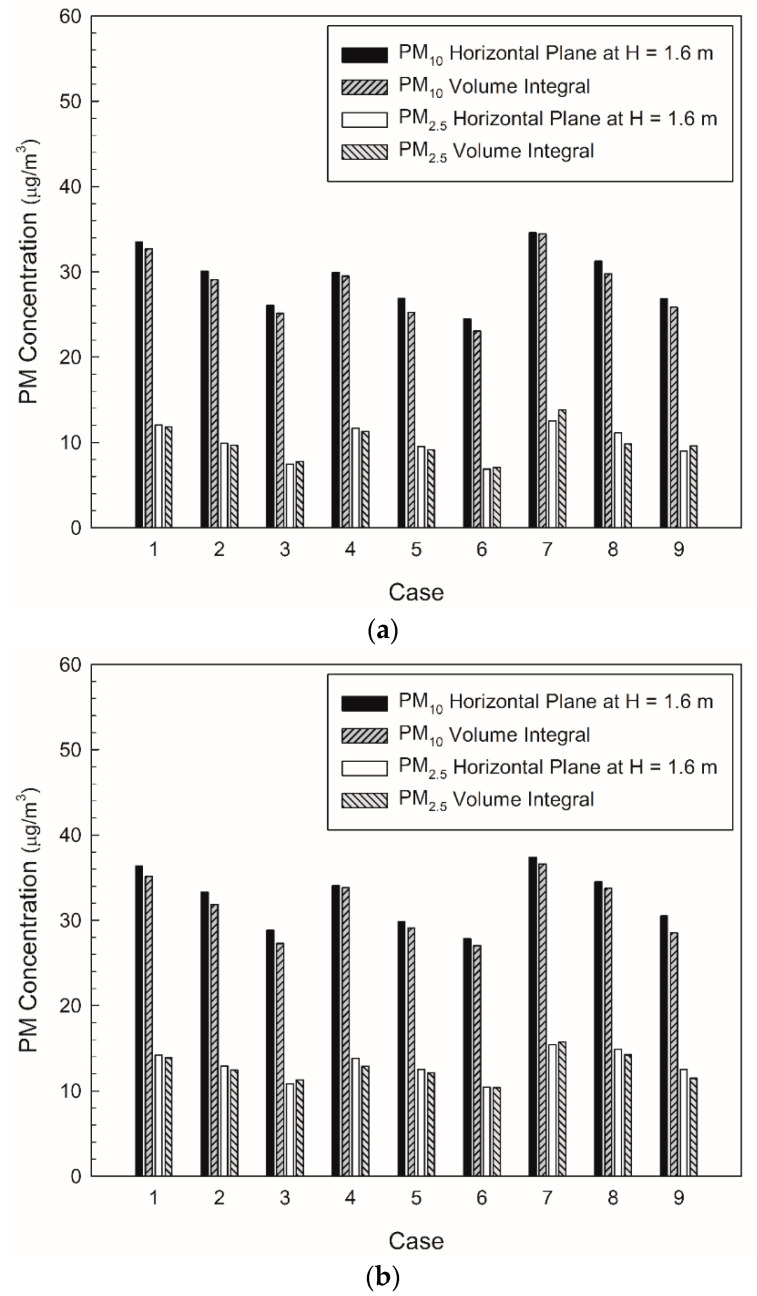
Comparison of average PM concentrations for each case: (**a**) waiting room (floor B1); (**b**) platforms (floor B2).

As examined above, the average PM_10_ and PM_2.5_ concentrations of station Y in the current situation were predicted to satisfy the stringent PM regulations of Seoul. However, certain local areas did not satisfy the criteria despite excluding the space close to the PSDs, where the airflow was introduced from the subway cabins. To address this problem, the PM concentrations were predicted by changing the filtration efficiency of the air-conditioning system and the diffuser arrangement in the waiting room, as indicated in Table 2; Figure 9 depicts the corresponding results. When the filtration efficiency was increased, the average PM concentrations in the subway station inherently decreased. When the same filtration efficiency was applied, the type B model exhibited the lowest average PM concentrations, followed by the type A and C models. In comparison with the results of the type A model, the PM_10_ and PM_2.5_ concentrations for the type B model, in which the flow rate of the diffusers located near the walls was increased, decreased by 10.5 and 5%, respectively. In the case of the type C model, in which the flow rate of the diffusers located in the central area was increased, the PM_10_ and PM_2.5_ concentrations were predicted to increase by 9 and 18%, respectively.

**Figure 10:**
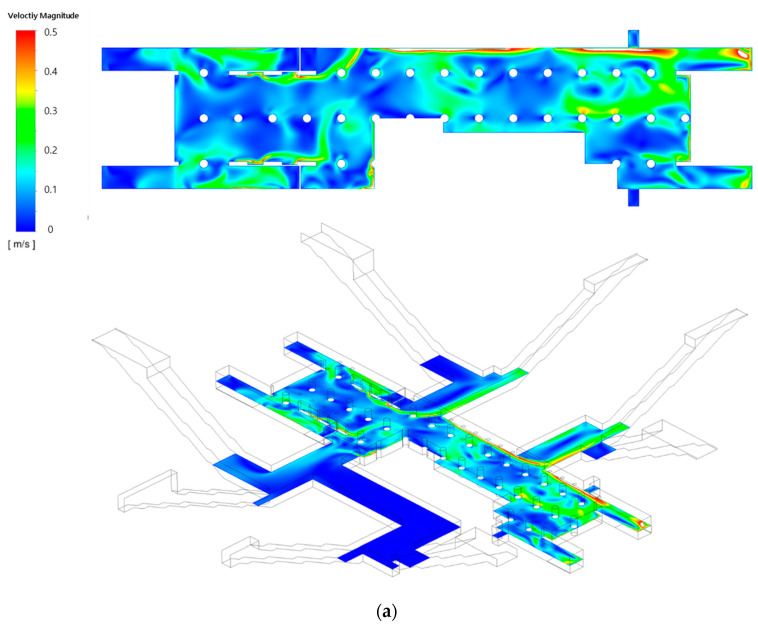

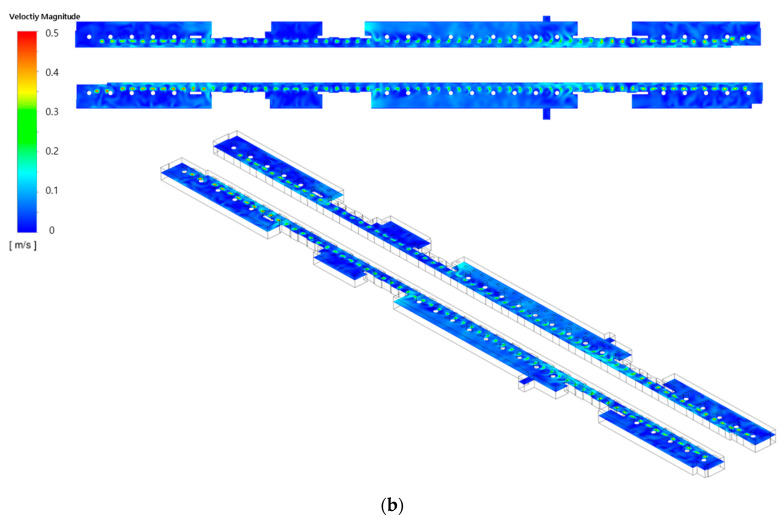
Airflow velocity distributions under the application of the type B diffuser arrangement: (**a**) waiting room (floor B1); (**b**) platforms (floor B2).

**Figure 11:**
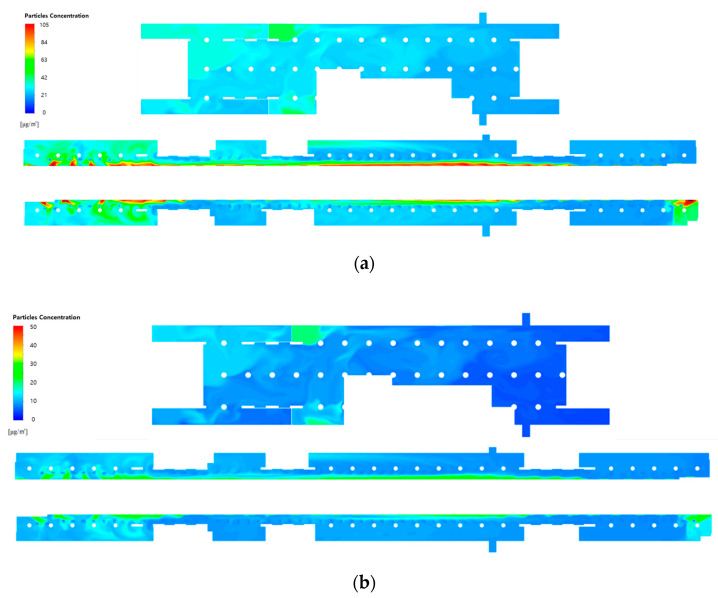
PM concentration contours in the waiting room (floor B1) and platforms (floor B2) under the application of type B diffuser arrangement at a filtration efficiency of 70% (Case 4): (**a**) PM_10_ concentration; (**b**) PM_2.5_ concentration.

Figure 10 and Figure 11 depict the airflow velocity and PM concentration distributions, respectively, at the height of the breathing line in the waiting room and platforms considering the type B diffuser arrangement at a filtration efficiency of 70% (Case 4). A comparison between Figure 6a and Figure 10a indicated that the airflow blown from the outside through the hallways connected to the ground entrances was blocked more effectively owing to the increase in the flow rate of the diffusers near the waiting room walls. This also affected the flow velocity distribution in the platforms connected through the stairs. When the velocity distribution of type B (Figure 10b) was compared with that of type A (Figure 6b), the flow in the platforms was determined to be more active in the former despite the diffuser arrangement remaining unchanged in the platforms. In the case of the type B diffuser arrangement, the average PM_10_ concentration in the waiting room was predicted to be 26.9 μg/m^3^ at the height of the breathing line and 26.5 μg/m^3^ in the entire space; whereas, the average PM_2.5_ concentration was predicted to be 11.7 μg/m^3^ at the height of the breathing line and 11.3 μg/m^3^ in the entire space. For the same diffuser arrangement (type B), the average PM_10_ concentration in the platforms was predicted to be 31.1 μg/m^3^ at the height of the breathing line and 30.8 μg/m^3^ in the entire space, while the average PM_2.5_ concentration was predicted to be 13.8 μg/m^3^ at the height of the breathing line and 12.9 μg/m^3^ in the entire space. Therefore, despite the identical total ventilation flow rate and filtration efficiency, the PM_10_ and PM_2.5_ concentrations in the waiting room and platforms decreased by approximately 10.5 and 5%, respectively, for Case 4 (type B diffuser arrangement (Figure 11)) compared to the results of Case 1 (type A diffuser arrangement (Figure 7)). The PM concentrations in the waiting room were low because the airflow from the hallways connected to the ground entrances was blocked more effectively owing to the increase in the flow rate of the type B diffuser arrangement near the walls. The results obtained in the case of the platforms were compared (Figure 7 and Figure 11), wherein the PM concentrations around the stairs connected to the first basement floor were determined to be reduced further. This is because the supply of the high flow rate from the diffusers near the walls around the stairs effectively blocked the airflow with high PM concentrations that ascended from the platforms along the stairs. Although high PM concentrations were inevitable in areas adjacent to opened PSDs because of the airflow introduced from subway cabins, the air quality on the platforms was improved despite the diffuser arrangement on the second basement floor remaining unchanged. Therefore, the type B diffuser arrangement was determined to be suitable for decreasing the PM concentrations in both the waiting room and platforms.

**Figure 12:**
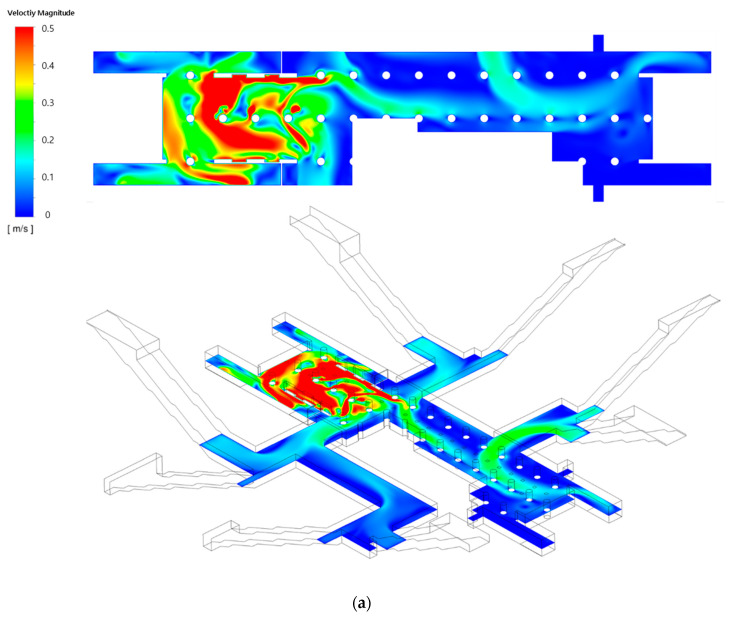

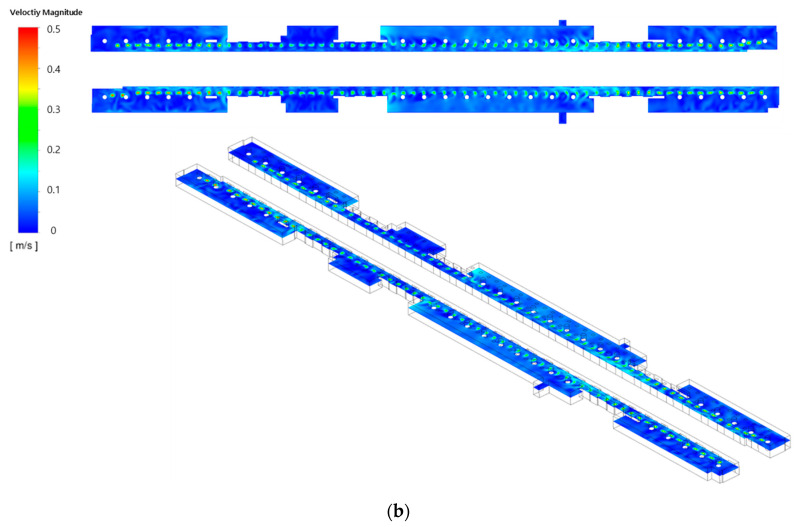
Airflow velocity distributions under the application of the type C diffuser arrangement: (**a**) waiting room (floor B1); (**b**) platforms (floor B2).

**Figure 13:**
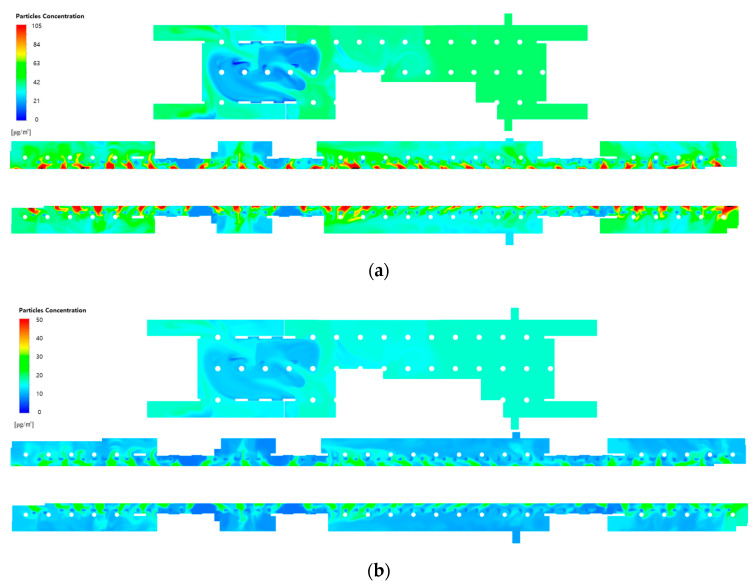
PM concentration contours in the waiting room (floor B1) and platforms (floor B2) under the application of type C diffuser arrangement at a filtration efficiency of 70% (Case 7): (**a**) PM_10_ concentration; (**b**) PM_2.5_ concentration.

Figure 12 and Figure 13 depict the airflow velocity and PM concentration distributions, respectively, at the height of the breathing line in the waiting room and platforms when the type C diffuser arrangement was applied at a filtration efficiency of 70% (Case 7). As indicated in Figure 12, the airflow introduced through the hallways connected to the ground was not effectively blocked because the diffusers in the waiting room were located only in the central area. Moreover, the outdoor air reached the platforms as it was blown to the stairs. In part A of the waiting room, the airflow with a relatively high speed stagnated and circled around the central area. Figure 13 indicates that the PM concentrations at the center of part A were significantly lower compared to other areas owing to the phenomenon of clean air, supplied from the diffusers in the central area of part A, circling around the space. However, the PM concentrations in areas other than the central area of part A were high because the airflow blown through the hallways connected to the ground entrances was not effectively blocked or diluted. Moreover, in the case of the type C model, diffusers were not placed near the stairs connected to the second basement floor; thus, the relatively clean air was not blown to the second basement floor. Furthermore, the airflow ascending from the second basement floor was not effectively blocked or diluted, resulting in higher PM concentrations in the entire space of the station, including the first and second basement floors, compared to that observed in type A and B models.

The obtained results verified that the diffuser arrangement significantly impacted the air quality inside the subway station despite the application of the same ventilation flow rate. Additionally, among the different types of diffuser arrangements considered in this study, the type B diffuser arrangement was identified as the most favorable for reducing the PM concentrations in the station to satisfy the stringent PM concentration criteria of Seoul. However, diffusers in the type B model were placed only near the walls of the waiting room. Therefore, further analysis is required in terms of thermal comfort during cooling and heating operations. In addition, the flow rate of the diffusers near the waiting room walls was increased for the type B model because the diffusers in the central area were not used. This warrants further research on the influence of an increase in the local flow velocity and noise. To derive satisfactory results for the impact of thermal comfort, appropriate flow velocity, and noise in addition to PM concentration conditions, a detailed parametric study should be performed by changing the flow rate ratio of the diffusers in the central area and near the waiting room walls. In summary, as the airflow introduced from the ground and subway cabins significantly impact the PM concentrations in the station, reducing the airflow from the ground and PM concentrations in subway cabins is important in addition to improving the air-conditioning systems in subway stations.

## 4. Conclusions

In this study, the PM concentrations in a subway station were analyzed and compared according to different air-conditioning diffuser arrangements and filtration efficiencies. In the case of the current air-conditioning system operating conditions of station Y, the PM_10_ and PM_2.5_ concentrations at the height of the breathing line were predicted to be 33.5 and 12.0 μg/m^3^ for the first basement floor, whereas they were 36.4 and 14.2 μg/m^3^ for the second basement floor, respectively. Both the PM_10_ and PM_2.5_ concentrations on the platforms on the second basement floor were determined to be higher than those on the first basement floor. This can be attributed to the introduction of airflow with high PM concentrations from the subway cabins when the PSDs were opened, and to the insufficient number of diffusers in the relatively large platform space. When the filtration efficiency was increased from 70 to 90% under the operating conditions of the current air-conditioning system, the average PM concentrations in the station were predicted to satisfy the stringent regulations of Seoul; however, the criteria were exceeded in certain local areas. Therefore, three models with different diffuser arrangements were considered in this study. The analysis indicated that the PM_10_ concentration in the waiting room on the first basement floor was decreased by approximately 10.5% and the PM_2.5_ concentration by approximately 5% for the type B model, wherein the flow rate of the diffusers near the waiting room walls was increased. However, the type C model with an increased flow rate of the diffusers in the central area of the waiting room exhibited a deterioration in the air quality in both the waiting room and platforms. This is because the airflow introduced from the ground entrances was not effectively blocked or diluted. When the diffuser arrangement of type B was applied while maintaining the current total ventilation flow rate of station Y, the stringent PM concentration criteria of Seoul were predicted to be satisfied in all local areas on the first and second basement floors despite a filtration efficiency of 70%; however, certain areas close to the opened PSDs failed to satisfy the regulations. Nevertheless, the obtained results confirmed that an appropriate diffuser arrangement can significantly improve the indoor air quality in subway stations despite the use of low-grade filters. In the future, systematic and detailed research on thermal comfort, noise, and appropriate flow velocity is required, in addition to the PM concentrations, based on the diffuser arrangement and flow rate of each diffuser. The results of this study can form the basis for deriving suitable measures for reducing the PM concentrations in subway stations.

## Figures and Tables

**Table 1:** Existing number of diffusers and flow rate conditions in each part of station Y.

Part			A	B	C	D	E	F	G	H	I	J
Input conditions	Supply	Number of diffusers	20	27	11	19	19	15	11	19	19	15
		Flow rate (m^3^/h)	979	843	530	340	410	560	650	420	380	527
	Return	Number of diffusers	20	24	11	8	17	11	11	8	17	11
		Flow rate (m^3^/h)	883	214	480	720	410	685	690	880	385	650

**Table 2:** Combinations of diffuser arrangement types and filtration efficiency in the waiting room.

	Floor B1 (Waiting Room) Diffuser Arrangement	Filtration Efficiency
Case 1	Type A	70%
Case 2	Type A	80%
Case 3	Type A	90%
Case 4	Type B	70%
Case 5	Type B	80%
Case 6	Type B	90%
Case 7	Type C	70%
Case 8	Type C	80%
Case 9	Type C	90%

## Data Availability

Not applicable.
